# Study on the Effects of Steel Slag on the Mechanical Properties and Microstructure of Cement-Stabilised Base Course Mixtures

**DOI:** 10.3390/ma19122539

**Published:** 2026-06-12

**Authors:** Shuyang Li, Yangpeng Zhang, Jin Li, Tianzhu Lan, Xiaodong Jiao

**Affiliations:** 1 Guangxi Transportation Science and Technology Group Co., Ltd., Guangxi Key Lab of Road Structure and Materials, Nanning 530007, China; 2 School of Civil Engineering and Architecture, Guangxi University, Nanning 530004, China

**Keywords:** cement-stabilised steel slag mixture, steel slag content, cement dosage, mechanical properties, microstructure

## Abstract

**Highlights:**

- The effects of cement dosage and steel slag content on the mechanical properties of cement-stabilised steel slag mixtures were investigated.
- Strength and modulus first increase with steel slag content and peak at 60% steel slag incorporation.
- Higher cement content improves the mechanical properties gradually with a declining growth rate.
- Appropriate cement and steel slag synergistically densify the microstructure and enhance mixture performance.

**Abstract:**

To address the environmental issues arising from the large-scale stockpiling of steel slag and to explore its efficient utilisation in road sub-bases, this study investigated the effects of cement dosage and steel slag content on the mechanical properties of cement-stabilised steel slag mixtures. Through unconfined compressive strength tests, compressive modulus of elasticity tests and splitting tensile strength tests, the study revealed the mechanisms by which cement dosage and steel slag content influence microstructure. The results indicate that as the cement content increases, the unconfined compressive strength, compressive modulus of elasticity and splitting tensile strength all show an upward trend, although the rate of increase gradually decreases. With increasing steel slag content, the unconfined compressive strength and splitting tensile strength first increase and then decrease slightly, whilst the compressive modulus of elasticity continues to rise. When 60% steel slag was incorporated, the 28-day unconfined compressive strength and splitting tensile strength reached their peak values, representing increases of 22.17% and 72.7% respectively compared to the control group. Further examination of the microstructure revealed that increasing the cement content and steel slag content enhances structural density and reduces surface porosity; however, excessive cement content and steel slag content have an adverse effect on mechanical properties. Consequently, the synergistic effect of an appropriate amount of steel slag and cement can significantly improve the mechanical properties and microstructure of the mixture. These findings are of great significance in promoting the green utilisation of solid waste materials, such as steel slag, in road engineering.

## 1. Introduction

Against the backdrop of efforts to accelerate high-quality development in the Western Land–Sea Economic Belt, road infrastructure construction in Guangxi is currently experiencing rapid growth. As a key raw material in road infrastructure construction, stone—a finite resource—has been subject to excessive exploitation during road-building processes. This has not only damaged the natural habitats of vegetation but has also led to environmental issues such as soil erosion and landslides, posing a serious threat to the healthy, harmonious and sustainable development of human society. To resolve the conflict between road infrastructure development and the need to preserve the green ecological environment amidst scarce natural resources, the search for an economical and environmentally friendly alternative to stone for use in road engineering has become a key research focus in the materials sector today.

Against the strategic backdrop of the nation’s accelerated development of the New Land–Sea Corridor in the West and its drive for high-quality development in the western region, Guangxi, as a key hub for integrated land–sea development, is witnessing rapid growth in the construction of road infrastructure, including motorways, national and provincial trunk roads, and rural roads. The scale of the road network continues to expand, and the total length of roads in service is steadily increasing. As the primary load-bearing layer of the road structure, the stability of the road sub-base directly determines the overall service life of the road and driving safety. Natural stone aggregates are the primary raw material for road sub-bases. However, with the implementation of large-scale transport infrastructure projects, natural sand and gravel resources have been subject to over-extraction, triggering a series of ecological and environmental issues, including the destruction of mountain vegetation, soil erosion, slope failures and ecological imbalances in river systems. At the same time, with natural aggregate reserves becoming increasingly scarce and extraction costs continuing to rise, traditional stone supply models are struggling to meet the long-term demands of large-scale transport construction in Guangxi. Against this backdrop, resolving the conflict between the massive demand for stone in road infrastructure, the scarcity of natural resources, and the need for ecological conservation has become a key research focus in the field of road engineering. This involves developing industrial solid waste-based road construction materials that are economical, low-carbon, and capable of serving as a scalable alternative to natural aggregates [1].

Industrial steel slag is a bulk alkaline solid waste generated during the steel smelting process. China’s annual stockpile of steel slag exceeds 100 million tonnes. As a major steel industry base in south-western China, Guangxi has a large volume of locally stockpiled steel slag and a relatively low rate of comprehensive utilisation. Long-term open-air storage not only occupies a significant amount of land resources but also poses environmental risks such as heavy metal leaching and alkaline seepage contaminating soil and groundwater, thereby constraining the development of the regional circular economy [2]. Existing research indicates that steel slag possesses characteristics such as high strength, low crush value, good wear resistance, excellent angularity and high alkaline reactivity. Its physical and mechanical properties are superior to those of some natural crushed stone, making it a highly promising green recycled aggregate [3]. When used in cement-stabilised base course materials, it can enhance strength, fill voids and optimise performance, thereby meeting the load-bearing, durability and stability requirements of road base courses. In recent years, scholars both domestically and internationally have conducted extensive exploratory research on the resource utilisation of steel slag and the performance of cement-stabilised steel slag base materials.

With regard to mechanical properties, numerous researchers have demonstrated the feasibility of using steel slag as a substitute for natural aggregate in cement-stabilised sub-bases [4,5]. Liu [6] et al. replaced crushed stone with steel slag on an equal weight basis to investigate the mechanical response of cement-stabilised crushed stone sub-bases at 30%, 50% and 70% replacement rates. They found that the strength and stiffness of the mixture were optimal at a 50% steel slag replacement rate; higher replacement rates deteriorated the overall performance due to the high porosity and high water absorption of steel slag. Zhou [7] et al. investigated the effect of steel slag content on the strength of cement–fly ash-stabilised steel slag mixtures, confirming that a reasonable increase in steel slag content can effectively enhance mechanical performance. Liu [6] et al. experimentally investigated the performance of base course mixtures with steel slag replacing aggregates at volume fractions of 30%, 50% and 70%. Mechanical and durability properties were tested, and micro-characterisation combined with molecular simulation was adopted to reveal the underlying mechanism. Results indicate that the mixture with 50% steel slag exhibits the optimal strength and stiffness, enhanced frost resistance and drying shrinkage, and reduced porosity, achieving the best overall performance with favourable economic and environmental benefits compared with limestone mixtures. Researchers have pointed out that the content of free calcium oxide and magnesium oxide in steel slag is a key factor affecting the long-term stability of cement-stabilised mixtures. An alkaline hydration environment can stimulate the latent cementitious activity of steel slag, generating hydration products such as C–S–H gel and ettrite, thereby achieving structural densification. Furthermore, recent cutting-edge research has increasingly focused on the interaction between steel slag, cementitious materials and the environment, investigating the effects of freeze–thaw cycles, salt corrosion, and temperature and humidity fluctuations on the long-term service performance of steel slag-based sub-bases, thereby providing a basis for the design of durable steel slag-based road materials [8,9].

Although existing research has confirmed the potential of steel slag for use in cement-stabilised sub-bases, there remain significant research limitations and shortcomings in terms of regional applicability. Firstly, the properties of steel slag exhibit significant regional variability. Physical indicators such as particle size distribution, crushing value and the content of acicular and flaky particles, as well as the mineralogical and chemical composition—including free CaO, free MgO and active SiO_2_—are directly influenced by regional steelmaking raw materials, smelting processes, cooling methods and the degree of weathering during stockpiling. Consequently, there are marked differences in the reactivity, expansion risk and cementitious properties of steel slag across different regions [6]. Most existing domestic and international studies have been conducted using steel slag from North China, East China, and imported sources. Consequently, the optimal blending ratios, mechanical behaviour patterns, and microstructural mechanisms identified in these studies are geographically limited and cannot be directly applied to locally sourced steel slag in Guangxi [10,11]. Secondly, there is a dearth of specialised research on the Guangxi region. The existing literature lacks systematic experiments that integrate Guangxi’s climate (high temperature and humidity and high rainfall), geological conditions, and the physicochemical properties of local steel slag. The cement dosage and optimal steel slag blending range suitable for Guangxi road sub-bases have not yet been clearly established, and there is a lack of localised performance regulation patterns and microstructural mechanism analyses. Consequently, it is unable to provide direct data and theoretical support for the engineering application of steel slag resource recovery in Guangxi.

To address the aforementioned research gaps, and based on the physicochemical properties of steel slag produced by local steel enterprises in Guangxi, this study focuses on cement-stabilised steel slag mixtures. Unconfined compressive strength, compressive modulus of elasticity, and splitting strength tests were conducted to systematically investigate the influence of cement dosage and steel slag content on the macroscopic mechanical properties of the mixtures; combined with microstructural observations, the study reveals the intrinsic mechanisms by which hydration products, mineral composition and micro-porosity influence macroscopic properties, thereby identifying the optimal cement-to-steel slag mix ratio suitable for the Guangxi region. This study will enrich the fundamental experimental data on the resource utilisation of steel slag in the region, refine the theoretical framework for regulating the performance of road base materials made from alkaline industrial solid waste under humid and hot conditions, promote the deep integration of large-scale steel slag utilisation with green road construction in Guangxi, and contribute to the high-quality, low-carbon and sustainable development of infrastructure along the New Land–Sea Corridor in the West. It will also provide technical references and practical evidence for the implementation of a regional circular economy [12,13].

## 2. Materials and Methods

### 2.1. Raw Materials

**Figure 1:**
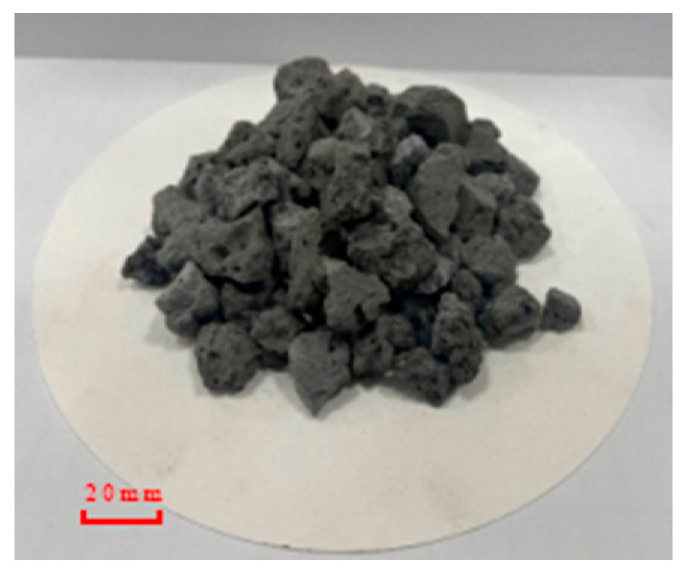
Steel slag aggregate.

The steel slag aggregate was sourced from unaged, hot-compacted steel slag produced by the Liuzhou Iron and Steel Group in Guangxi, as shown in Figure 1. The steel slag particles are deep black in colour and exhibit a highly porous surface structure. The physical properties of the steel slag are shown in Table 1. The slag has a crushing value of 17.5% and a water-soaked expansion rate of 0.72%, meeting the requirements of the standard ‘Steel Slag for Road Construction’ (GB/T 25824-2010) [14].

The main chemical composition of this steel slag is shown in Table 2. The primary constituents are CaO, SiO_2_, MgO, Fe_2_O_3_, Al_2_O_3_, etc., accounting for a total of 88%. The pH of steel slag is calculated using the following formula [15]:

$$M_{1}=\frac{w\left(MgO\right)+w\left(CaO\right)+w\left(Al_{2}O_{3}\right)}{w\left(SiO_{2}\right)}$$

$$M=\frac{w\left(MgO+CaO\right)}{w\left(SiO_{2}\right)+w\left(Al_{2}O_{3}\right)}$$

In the equation: *w* represents the content of the mineral component. A value of *M*_1_ < 1 indicates an acidic mineral, whilst a value of *M*_2_ > 1 indicates an alkaline mineral. The calculated values are 4.06 for *M*_1_ and 1.85 for *M*_2_; therefore, this steel slag aggregate is classified as an alkaline aggregate, indicating that the steel slag has a high level of alkali reactivity.

The cement used in this study was P·O 42.5 ordinary Portland cement; its performance parameters are shown in Table 3. The manufactured limestone sand was sourced from a sand quarry in Nanning, Guangxi.

### 2.2. Mixture Designs

**Figure 2:**
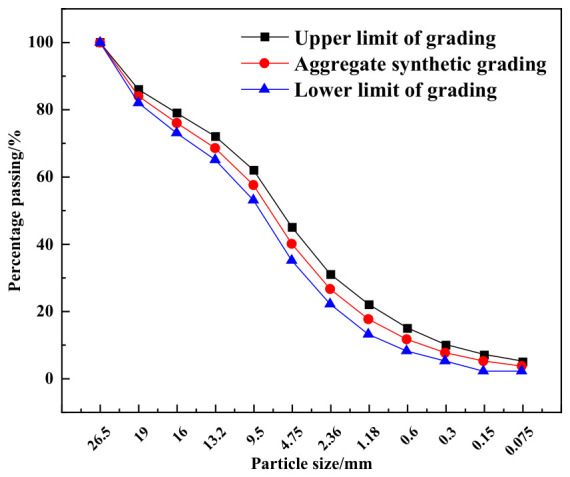
Grading curve.

According to the recommended cement dosages (3%, 4%, 5%, 6%, and 7%) for mix proportion tests of the cement-stabilised materials listed in Table 4.6.4 of the Technical Guidelines for Construction of Highway Pavement Bases (JTG/T F20-2015) [16], preliminary trial tests were conducted. The results indicate that cement-stabilised steel slag mixtures can satisfy the strength requirements of the specification and maintain specimen integrity when the cement dosage is 3–4.5% and the steel slag content ranges from 20% to 80%. Combined with existing research results of previous scholars [6], four cement dosages (3%, 3.5%, 4%, and 4.5%) and five steel slag contents (0%, 20%, 40%, 60%, and 80%) were selected for mix proportion design in this study, generating nine different mix proportions of cement-stabilised steel slag mixtures. The median gradation of C-B-3 recommended in JTG/T F20-2015 [16] was adopted, and the gradation curve is shown in Figure 2.

### 2.3. Test Methods

#### 2.3.1. XRD and SEM Tests of Steel Slag

The crystal structure of steel slag was analysed using a Rigaku D/MAX 2500V X-ray diffractometer (Tokyo, Japan). The steel slag particles were placed in a drying oven at 60 °C for two days. After drying, the particles were ground into a powder, sieved through a 45 μm sieve, and then evenly distributed into a sample mould ring and compacted. The data was then analysed using the X-ray diffractometer. The scanning speed of the X-ray diffractometer was 10°/min, with a diffraction angle 2θ ranging from 10° to 90°.

The microstructure and morphology of the steel slag were examined and analysed using an S-3400N scanning electron microscope (Hitachi, Tokyo, Japan). The dried steel slag was crushed into pieces of approximately 1 cm^2^, and the samples were affixed to a copper specimen holder using conductive adhesive. The surface of the specimens was treated with a gold coating, after which the samples were examined and analysed under the scanning electron microscope.

#### 2.3.2. Compaction Test of Mixtures Containing Steel Slag

**Figure 3:**
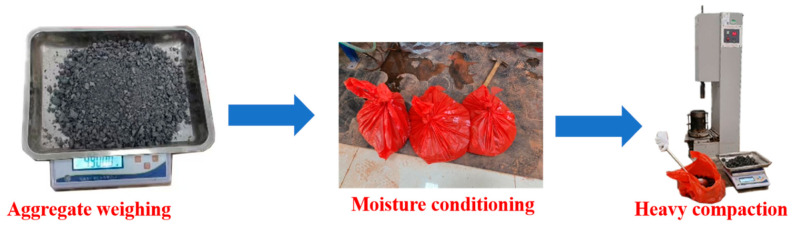
Compaction test procedure.

In accordance with the ‘Test methods of materials stabilised with inorganic binders for highway engineering’ (JTG3441-2024) [17], compaction tests were conducted on the nine different aggregate gradations listed in Table 4. Figure 3 shows compaction test of mixtures containing steel slag. The tests were carried out in three layers, with each layer compacted 98 times, to determine the optimum moisture content and maximum dry density for each aggregate mix.

#### 2.3.3. Mechanical Property Tests of Mixtures Containing Steel Slag

**Figure 4:**
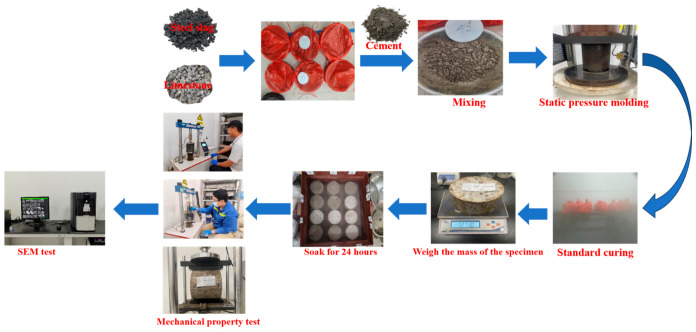
Test flowchart for characteristics of mixtures containing steel slag.

Based on the optimum moisture content and maximum dry density obtained from the compaction tests, static compaction was carried out in accordance with the ‘Test Procedures for Inorganic-Bound Stabilised Materials in Highway Engineering’ (JTG3441-2024) [17]. To ensure the reliability and accuracy of the test results, each set of specimens comprised no fewer than 13 test pieces. Figure 4 shows test flowchart for characteristics of mixtures containing steel slag. The mould dimensions were φ100 mm × 100 mm. After the mixture was demoulded, its height was measured and its weight recorded, and it was then placed in a plastic bag. The air was thoroughly expelled from the bag, the opening was tightly sealed, and the wrapped specimen was placed in the curing chamber. The standard curing temperature was 20 °C ± 2 °C, and the standard curing humidity was ≥95%. The specimens are placed on racks with a spacing of at least 10–20 mm. On the final day of the curing period, the specimens are removed, their height is measured and their mass weighed, and they are then immersed in water at 20 °C ± 2 °C, ensuring that the water level is approximately 25 mm below the top surface of the specimens. After 24 h of immersion, unconfined compressive strength, compressive modulus of elasticity and splitting strength tests were conducted, yielding the 7-day, 28-day and 60-day unconfined compressive strength, compressive modulus of elasticity and splitting strength for mixtures with different mix proportions (Figure 4).

#### 2.3.4. SEM Test of Steel Slag Mixtures Containing Steel Slag

After testing their mechanical properties, the broken specimens were placed in anhydrous ethanol and soaked for three days to complete hydration and then dried in an oven at 60 °C for two days. Following drying, the specimens were gold-sputtered, and their interface transition zones, microstructure and morphology were examined using an S-3400N scanning electron microscope.

## 3. Results and Discussion

### 3.1. Results of Compaction Test

**Figure 5:**
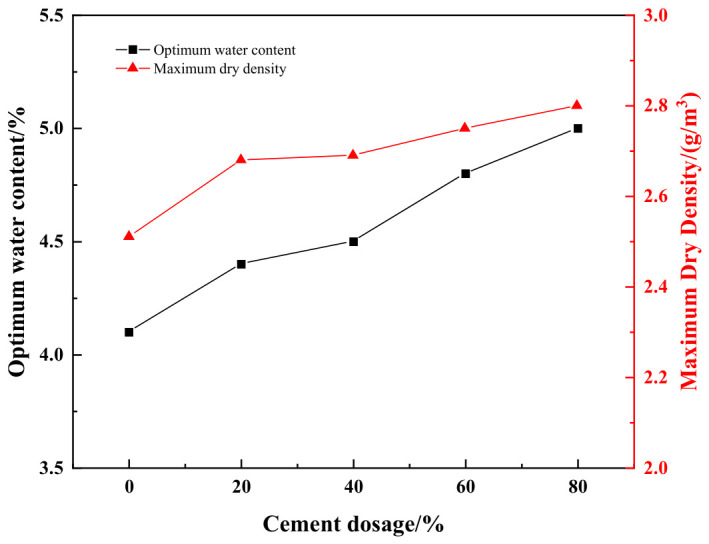
Results of gradation compaction tests with different steel slag content.

Figure 5 shows the results of gradation compaction tests for different steel slag content levels. As can be seen from the figure, both the optimum moisture content and maximum dry density increase as the steel slag content rises. The mixture comprising 80% steel slag aggregate and 20% limestone aggregate exhibited the highest optimum moisture content and maximum dry density, at 5% and 2.8 g/cm^3^ respectively. Steel slag has a high surface porosity and a higher water absorption rate compared to limestone aggregate; incorporating a greater proportion of steel slag aggregate increases the optimum moisture content. Furthermore, as the density of steel slag is significantly greater than that of limestone, the maximum dry density of the mixture shows an increasing trend with an increase in the steel slag content [18].

### 3.2. Analysis of Mechanical Properties

#### 3.2.1. Unconfined Compressive Strength

**Figure 6:**
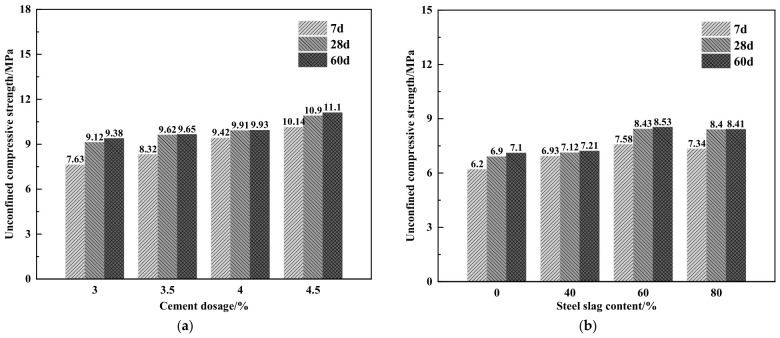
Effect of various factors on unconfined compressive strength: (**a**) cement dosage; (**b**) steel slag content.

The patterns of how cement dosage and steel slag content affect the unconfined compressive strength of cement-stabilised steel slag mixtures are shown in Figure 6. As can be seen from Figure 6, as the cement dosage increases from 3% to 4.5%, the unconfined compressive strength of the cement-stabilised steel slag mixture at 7, 28 and 60 days shows a steadily increasing trend; when the cement dosage is 3%, the unconfined compressive strengths at 7, 28 and 60 days reached 7.63 MPa, 9.12 MPa and 9.38 MPa respectively. The 7-day unconfined compressive strength met the requirements for sub-bases under extremely heavy and special heavy traffic loads on expressways and Class I highways, as specified in the ‘Technical Specifications for Construction of Highway Pavement Sub-bases’ (JTG/T F20-2015). As the cement content increased from 3% to 4.5%, the 7-day, 28-day and 60-day unconfined compressive strengths rose to 10.14 MPa, 10.9 MPa and 11.1 MPa, representing increases of 33.15%, 16.33% and 18.33% respectively compared to the 3% cement content. Steel slag forms a well-compacted structure with the hydration products generated during cement hydration and effectively fills the voids within the mixture; as the degree of compaction increases, so does the strength [19]. As shown in Figure 6, as the steel slag content increases, the unconfined compressive strength of the cement-stabilised steel slag mixture at 7, 28 and 60 days exhibits a trend of initially rising and then slightly decreasing. When 40% steel slag was incorporated, the unconfined compressive strengths at 7, 28 and 60 days reached 6.93 MPa, 7.12 MPa and 7.21 MPa respectively. Compared with ordinary cement-stabilised crushed stone mixtures, the unconfined compressive strengths at 7, 28 and 60 days increased by 11.77%, 3.18% and 1.55% respectively. When the steel slag content was increased to 60%, the unconfined compressive strengths at 7, 28 and 60 days reached their maximum values of 7.58 MPa, 8.43 MPa and 8.53 MPa. As the steel slag content was further increased to 80%, the unconfined compressive strengths at 7, 28 and 60 days decreased slightly, falling by 3.16%, 0.36% and 1.40% respectively compared to the cement-stabilised steel slag mixture with a 60% steel slag content. This is because the higher porosity of the steel slag adsorbs a greater amount of cement paste, which affects the hydration reaction of the cement and consequently has an adverse effect on the development of unconfined compressive strength [20].

#### 3.2.2. Compressive Modulus of Elasticity

**Figure 7:**
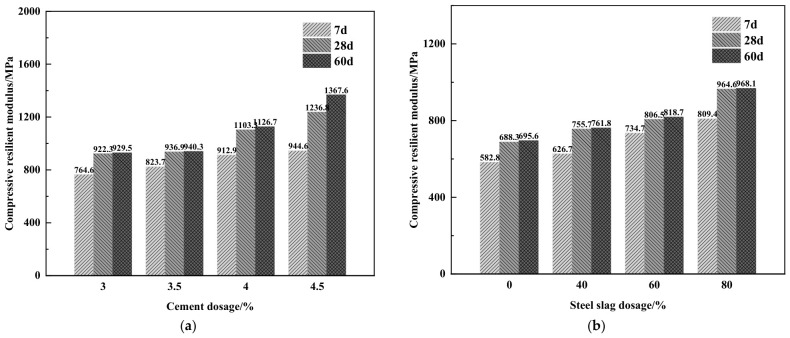
Effect of various factors on compressive modulus of elasticity: (**a**) cement dosage; (**b**) steel slag content.

The patterns of how cement dosage and steel slag content affect the compressive rebound modulus of cement-stabilised steel slag mixtures are shown in Figure 7. Figure 7a reveals that as the cement dosage increases from 3% to 4.5%, the compressive rebound modulus of the cement-stabilised steel slag mixture at 7, 28 and 60 days shows a steadily increasing trend. As the cement content increased from 3% to 4.5%, the compressive rebound modulus at 7, 28 and 60 days increased by 23.54%, 34.1% and 47.13% respectively. This is because, at lower cement contents, the aggregates are not adequately bonded together, resulting in poor mix integrity, with the steel slag aggregate skeleton acting as the primary load-bearing structure. As the cement content increased to 4.5%, the cement was able to effectively bind the steel slag aggregates together, thereby enhancing the load-bearing capacity of the cement-stabilised steel slag mixture [21]. As shown in Figure 7b, the relationship between the steel slag content and the compressive rebound modulus of cement-stabilised steel slag follows the same pattern as that observed with cement dosage. The compressive rebound moduli of ordinary cement-stabilised crushed stone mixtures at 7 days, 28 days and 60 days were 582.8 MPa, 688.3 MPa and 695.6 MPa, respectively. With the incorporation of 80% steel slag aggregate, the compressive rebound modulus increased significantly, with the compressive rebound modulus at 7 days, 28 days and 60 days increasing by 38.88%, 40.14% and 39.18% respectively. Compared to limestone, steel slag has a lower crushing value and higher load-bearing capacity; consequently, the compressive rebound modulus of the mixture continues to increase as the steel slag content rises [22].

#### 3.2.3. Splitting Tensile Strength

**Figure 8:**
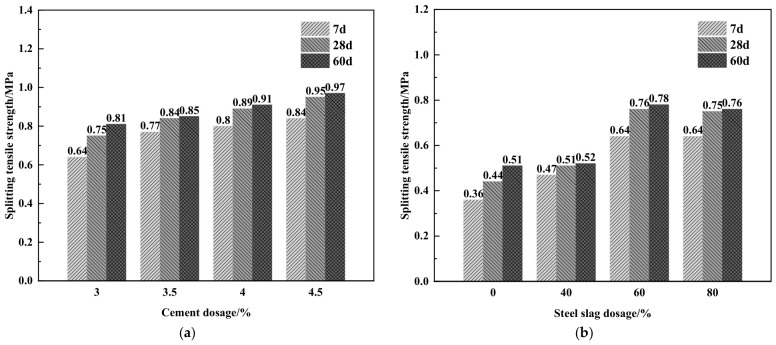
Effect of various factors on splitting tensile strength: (**a**) cement dosage; (**b**) steel slag content.

Figure 8 illustrates the effect of different cement contents and steel slag additions on the splitting tensile strength of cement-stabilised steel slag mixtures. As shown in Figure 8a, when the cement content increased from 3% to 4.5%, the 7-day, 28-day and 60-day splitting tensile strength of the cement-stabilised steel slag aggregate increased progressively. When the cement content was increased from 3% to 4.5%, the 7-day, 28-day and 60-day splitting tensile strength increased by 31.25%, 26.67% and 19.75% respectively. An increase in the cement content has a positive effect on the splitting tensile strength of cement-stabilised steel slag mixtures. This is because a higher cement content effectively binds the aggregate particles together, enhancing the interparticle bonding force and thereby improving the mixture’s resistance to splitting [23]. As shown in Figure 8b, as the steel slag content increases, the trend in splitting tensile strength at 7, 28 and 60 days initially rises before levelling off. The 7-day and 28-day splitting tensile strength of ordinary cement-stabilised crushed stone mixtures were 0.36 MPa and 0.44 MPa, respectively. When 60% steel slag was incorporated, the splitting tensile strength improved to a certain extent, with the 7-day and 28-day splitting tensile strength increasing by 77.78% and 72.72% respectively. As the steel slag content increased to 80%, there was little change in the splitting tensile strength at 7, 28 and 60 days. As splitting tensile strength depends on the bonding force between particles, increasing the steel slag content has little effect on splitting tensile strength.

### 3.3. Analysis of Micro-Mechanisms

#### 3.3.1. Analysis of the Mineral Composition and Microstructure of Steel Slag

**Figure 9:**
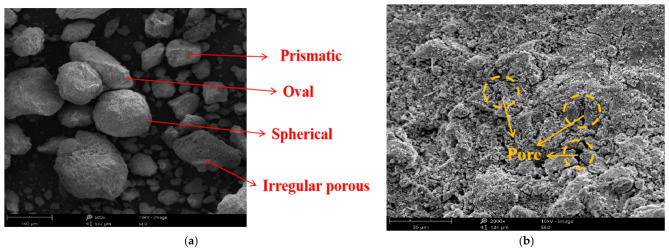
Microscopic morphology of steel slag: (**a**) 500× image; (**b**) 2000× image.

Analysis in Figure 9 shows that steel slag contains a relatively large number of complex mineral phases, primarily Ca_2_SiO_4_ and Ca_3_SiO_5_. Consequently, steel slag possesses a certain degree of hydration potential and, under specific conditions, can form hydration products such as calcium silicate hydrate. This facilitates the formation of such hydration products with cement, whilst simultaneously enhancing the bond strength with the cement paste and aggregate, thereby improving the mechanical properties of cement-stabilised steel slag mixtures [24].

A microscopic morphological analysis of steel slag aggregate reveals, upon examination of a 500× magnified image, that the particles exhibit a complex and diverse morphology, including spherical, elliptical, prismatic and irregular shapes; compared to ordinary crushed stone, they are less angular. Upon further magnification to 2000×, it can be observed that the surfaces of the steel slag particles are covered with numerous pores of varying sizes. This abundance of pores enhances the interlocking and mutual adsorption capabilities between steel slag particles and between steel slag and crushed stone and further facilitates a tight bond between the steel slag and the cement paste, thereby contributing to improved mechanical properties of the cement-stabilised steel slag mixture.

#### 3.3.2. Analysis of the Microstructure of Cement-Stabilised Steel Slag Mixtures

**Figure 10:**
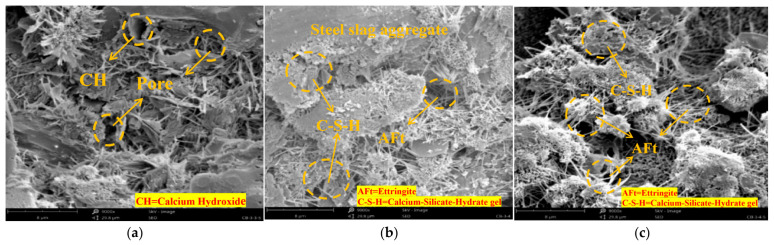
Microstructure of cement-stabilised steel slag test specimens with varying cement dosages: (**a**) steel slag mixture with cement dosages of 3.5%; (**b**) steel slag mixture with cement dosages of 4%; (**c**) steel slag mixture with cement dosages of 4.5%.

**Figure 11:**
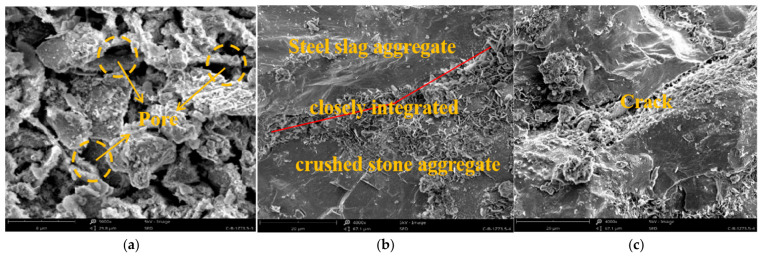
Microstructure of cement-stabilised steel slag test specimens with varying steel slag content: (**a**) steel slag mixture with steel slag content of 0%; (**b**) steel slag mixture with steel slag content of 60%; (**c**) steel slag mixture with steel slag content of 80%.

To elucidate the mechanisms by which cement dosage and steel slag content affect the microstructure of cement-stabilised steel slag mixtures, scanning electron microscopy tests were conducted; the results are shown in Figure 10 and Figure 11 below.

Figure 10 shows the microstructure of cement-stabilised steel slag mixtures with three different cement dosages. As can be seen from Figure 10a, hydration products such as calcium silicate hydrate, generated by the cement hydration reaction, fill the voids in the steel slag aggregate and bridge the gaps between the steel slag aggregates, thereby forming a cohesive whole. However, it can be observed that significant voids still exist within the steel slag aggregate framework, with a limited amount of hydration products, which has an adverse effect on the overall strength of the cement-stabilised steel slag aggregate mixture. Furthermore, the microstructure reveals that the overall structure contains numerous voids of varying sizes, with insufficient bonding between particles; the cementitious matrix has not yet fully filled the minute voids between the particles. As shown in Figure 10b, as the cement dosage increases to 4%, the cement hydration reaction intensifies, and the quantity of hydration products, such as calcium silicate hydrate, increases, densely filling the voids within the steel slag aggregate. Compared to the cement-stabilised steel slag aggregate with a cement content of 3.5%, the pore area between the steel slag aggregates shows a decreasing trend, with a reduction in the number of pores, which is more conducive to improving the mechanical strength of the cement-stabilised steel slag mixture. Zhang [25] et al. also found in their research that an increase in cement content facilitates the formation of hydration products, thereby strengthening the overall structure of the mixture and continuously enhancing its mechanical properties. When the cement content was increased to 4.5%, the microstructure of the cement-stabilised steel slag aggregate showed a slight improvement compared to that with a cement content of 4%, but the extent of the change was not significant. This is because, when the cement content is too high, pores on the surface of the steel slag particles can adsorb excess cement, thereby affecting the cement hydration reaction; consequently, the improvement in the microstructure of the cement-stabilised steel slag aggregate is limited [26].

Figure 11 shows the microstructures of cement-stabilised steel slag mixtures with three different steel slag contents. Figure 11a depicts the microstructure of a standard cement-stabilised crushed stone mixture. It can be observed that the bond between the cement hydration products and the crushed stone is poor; the hydration products have failed to form a cohesive whole with the crushed stone matrix, resulting in a relatively loose structure with numerous voids. When 60% steel slag was incorporated, the bond between the cement hydration products and the steel slag aggregate was significantly stronger. The cement-stabilised steel slag mixture exhibited greater structural integrity, with no obvious voids or pore structures observed. The interface transition zone was filled with hydration products, indicating good structural integrity and the formation of a dense, cohesive structure. Wang et al. [27] also noted that, following the incorporation of steel slag, the hydration products were distributed more uniformly in the interface transition zone, and the quantity of hydration products such as calcium silicate hydrate increased significantly. As the steel slag content increased to 80%, micro-cracks appeared in the structure and the number of voids and pores increased; the interface transition zone became a weak point in the overall structure [28]. Consequently, the mechanical properties of the cement-stabilised steel slag mixture with an 80% steel slag content showed a slight decline.

## 4. Conclusions

(1) As the cement content increased, the unconfined compressive strength, compressive modulus of elasticity and splitting tensile strength of the cement-stabilised steel slag mixture showed a rising trend, although the rate of increase gradually decreased. With a steel slag content of 80%, when the cement content was increased to 4%, the 28-day unconfined compressive strength, compressive modulus of elasticity and splitting tensile strength were 9.91 MPa, 1103.3 MPa and 0.89 MPa, respectively. As the cement content was further increased to 4.5%, the 28-day unconfined compressive strength, compressive modulus of elasticity and splitting tensile strength increased slightly, by only 2.27%, 10.79% and 6.74% respectively.

(2) As the steel slag content increased, the unconfined compressive strength and splitting tensile strength of the cement-stabilised steel slag mixture showed a trend of initially increasing and then slightly decreasing, whilst the compressive modulus of elasticity continued to rise. When 60% steel slag aggregate was incorporated, the 28-day unconfined compressive strength and splitting tensile strength of the mixture were 8.43 MPa and 0.76 MPa respectively, representing increases of 22.17% and 72.7% compared to ordinary cement-stabilised crushed stone mixtures. As the steel slag content increased to 80%, the unconfined compressive strength and splitting tensile strength showed a slight decrease, falling by 0.36% and 1.3% respectively.

(3) As the cement dosage and steel slag content increased, the quantity of hydration products rose. More hydration products filled the spaces between the steel slag aggregate and the cement paste, enhancing the density of the interface transition zone and the paste, which further densified the microstructure of the mixture. Consequently, the mechanical properties of the cement-stabilised steel slag mixture were further improved. However, when the cement content was excessive, the pores in the steel slag adsorbed excess cement, which impeded the normal hydration reaction of the cement, resulting in only a limited improvement in mechanical properties.

## Figures and Tables

**Table 1:** Physical properties of steel slag.

Bulk Specific Gravity/g/cm^3^	Water Absorption/%	Crushing Value/%	Flaky and Elongated Particle Content/%	Silt Content/%	Soft Stone Content/%	Immersion Swelling Rate/%
3.315	1.25	17.5	0.8	0.1	0.7	0.72

**Table 2:** Chemical composition of steel slag/%.

SiO_2_	CaO	MgO	Fe_2_O_3_	Na_2_O	P_2_O_5_	Al_2_O_3_	MnO	Others
15.14	38.75	11.07	11.30	0.90	1.06	11.74	1.37	8.67

**Table 3:** Physical properties of cement.

Standard Consistency/%	Initial and Final Setting Times (min)		Compressive Strength/MPa		Flexural Strength/MPa		Fineness/%	Density/(g/cm^3^)
	Initial	Final	3 d	28 d	3 d	28 d		
26	200	335	30.8	51.9	5.0	7.2	10.9	3.051

**Table 4:** Mix design for cement-stabilised steel slag mixture.

Serial Number	Mass Fraction/%		
	Cement	Steel Slag	Limestone
1	3	80	20
2	3.5	80	20
3	4	80	20
4	4.5	80	20
5	3.5	0	100
6	3.5	20	80
7	3.5	40	60
8	3.5	60	40
9	3.5	80	20

## Data Availability

The original contributions presented in this study are included in the article. Further inquiries can be directed to the corresponding authors.
